# Therapeutic inhibition of microRNA-21 (miR-21) using locked-nucleic acid (LNA)-anti-miR and its effects on the biological behaviors of melanoma cancer cells in preclinical studies

**DOI:** 10.1186/s12935-020-01394-6

**Published:** 2020-08-10

**Authors:** Shaghayegh Haghjooy Javanmard, Golnaz Vaseghi, Ahmad Ghasemi, Laleh Rafiee, Gordon A. Ferns, Hajar Naji Esfahani, Reza Nedaeinia

**Affiliations:** 1 Applied Physiology Research Center, Cardiovascular Research Institute, Isfahan University of Medical Sciences, Isfahan, Iran; 2 Isfahan Cardiovascular Research Center, Cardiovascular Research Institute, Isfahan University of Medical Sciences, Isfahan, Iran; 3 Department of Medical Education, Brighton and Sussex Medical School, Falmer, Brighton, BN1 9PH Sussex UK; 4 Pediatric Inherited Diseases Research Center, Research Institute for Primordial Prevention of Non-Communicable Disease, Isfahan University of Medical Sciences, Isfahan, Iran

**Keywords:** Cutaneous melanoma, miRNAs, miR-21, LNA-anti-miR-21

## Abstract

**Background:**

Melanoma is a cancer that has a high mortality rate in the absence of targeted therapy. Conventional therapies such as surgery, chemotherapy, and radiotherapy are associated with poor prognosis. The expression of miR-21 appears to be of clinical importance, and the regulation of its expression appears to be an opportunity for treatment.

**Methods:**

In this current study, we aimed to evaluate the effects of miR-21 inhibition in- vitro and in-vivo. In-vitro studies have investigated LNA-anti-miR-21 in mouse melanoma cells (B16F10), and in-vivo studies have proposed a model of melanoma in male C57BL/6 mice. To evaluate the anticancer effects of LNA-anti-miR-21, a QRT-PCR analysis was performed using the 2^−ΔΔCT^ method to determine the degree of inhibition of oncomiR-21. The MTT test, propidium iodide/AnnexinV in-vitro, and tumor volume measurement using the QRT-PCR test with the 2^−ΔΔCT^ method were used to estimate the inhibition of miR-21 and the expression of downstream genes including: *SNAI1, Nestin (Nes), Oct-4*, and *NF-kB* following miR-21 inhibition. Finally, immunohistochemistry was conducted for an in-vivo animal study.

**Results:**

MiR-21 expression was inhibited by 80% after 24 h of B16F10 cell line transfection with LNA-anti-miR-21. The MTT test showed a significant reduction in the number of transfected cells with LNA-anti-miR-21. The transfected cells showed a significant increase in apoptosis in comparison with the control and scrambled LNA groups. According to our in vivo findings, anti-miR-21 could reduce tumor growth and volume in mice receiving intraperitoneal anti-miR after 9 days. The expression of the *SNAI1*gene was significantly reduced compared to the controls. Immunohistochemical analysis showed no change in *CD133* and *NF-kB* markers.

**Conclusion:**

*Our* findings suggest LNA-anti-miR-21 can be potentially used as an anticancer agent for the treatment of melanoma.

## Background

Melanoma is a malignant cancer affecting cells containing a pigment, known as melanin, and predominantly affects the skin. Skin cancer, together with squamous cell carcinoma and basal cell carcinoma, is among the most prevalent cancers [1]. Although melanoma only accounts for about 1% of skin cancers, it is responsible for the majority of deaths from skin cancer [1]. According to the American Cancer Society, approximately 100,350 new melanomas (about 60,190 men and 40,160 women) are predicted in the United States by 2020. It seems that about 6850 people have died due to melanoma (about 4610 men and 2240 women) in the United States in  2020. Over  the past few decades, the prevalence of melanoma has increased [1]. The risk of melanoma increases with age. Although the average age of patients with melanoma is 65 years, its incidence is still high among individuals under 30 years of age; therefore, it is recognized as one of the most prevalent cancers in the young, particularly women [1]. Despite significant advances in treatment, malignant melanoma (MM) has a poor prognosis [2]. Drug resistance is usually related to the abnormal expression of apoptosis molecules, including *FLIP, Bcl-2, Bcl-XL, MCL-1, P53, Bax*, and *FADD* [3].

Drugs commonly used in chemotherapy for melanoma include cisplatin and oxaliplatin, which are not very effective and there is increasing prevalence of resistance to treatment [4]. One of the current chemotherapy methods is 5-fluorouracil (5-FU) along with capecitabine, targeting sodium thymidylate (TS) and thymidine monophosphate enzymes. However, their application is limited by the excessive expression of tumor thymidylate synthase following treatment with 5-FU and other thymidylate synthase inhibitors [5]. Other drugs used in chemotherapy include: temozolomide (TMZ) and dacarbazine (DTIC), but their overall success in preventing melanoma metastasis is very limited [6]. Similarly, DTIC, an FDA approved chemotherapy for melanoma, does not enhance the overall survival (OS). A relatively frequent finding in melanoma is resistance to alkylated agents, as well as the increased expression of O^6^-alkylguanine DNA alkyltransferase (MGMT) [7]. Cancer cells proliferate at a high rate and have poor restorative mechanisms; hence, they are more sensitive to DNA damage. However, anti-proliferating cellular alkylating agents are cytotoxic for normal divided cells. For instance, the testicles, bone marrow, mucous, and ovarian cells can result in complications, such as infertility. Moreover, the majority of alkaline agents are carcinogenic and involved in secondary malignancies [8, 9].

MicroRNAs (miRNAs) are small non-coding RNAs with a size of 25–19 nucleotides, playing a major role in various biological and pathologic processes. They are known as gene expression regulators after transcription, which inhibit the translation or breakdown of target mRNAs through specific sites linked to 3′-UTR in the target mRNAs [10]. A particular miRNA can communicate with hundreds of different mRNAs, which are estimated to control more than 30% of total proteins, encoded by the human genome. According to several studies, miRNAs, such as miR-15b, miR-204, miR-331, miR-342, miR-367, miR-622, miR-612, and let-7b, contribute to the progression of melanoma [11–14]. Recently, research has shown that many beneficial medications in the treatment of melanoma have their own effects by changing the expression of miRNAs. For example, metformin strongly suppresses the growth of melanoma cancer cells by causing cell cycle arrest and increasing cell apoptosis during the G2/M phase. Three miRNAs, i.e., miR-584-3p, miR-192-5p, and miR-1246, are highly recognized in metformin-treated melanoma cancer cells [11]. Studies show that miR-192-5p and miR-584-3p can stronglysuppress melanoma cell metastasis [11]. Therefore, a targeted treatment is different from standard chemotherapy that affects all rapidly dividing cell. Targeted therapy is more specific affecting cancer cells exclusively. The agents used in targeted therapy target molecules that grow and spread the tumor. MiRNAs have recently attracted major attention concerning the study of molecular pathways involved in cancer. However, there is no general agreement as to which miRNAs should be selected as biomarkers [15]. Studies have reported miR-21 expression in different cancers [16]. It plays a role in proliferation, invasion, metastasis, and angiogenesis by affecting and increasing the stemness properties of the cancer cells [17]. Inhibition of miRNAs is an opportunity for the appropriate treatment of certain cancers. In this regard, antisense oligonucleotides, such as LNA, are suitable alternatives for entry into the cell using appropriate gene transfer techniques [18]. These oligonucleotides do not generate immune responses and are non-toxic and stable; hence, they are used as a post-transcriptional gene silencing agent based on antisense gene therapy [18].

In this current study, we aimed to evaluate the effects of miR-21 inhibition (miRCURY LNA inhibitor®) in the B16F10 melanoma cell line in vitro and C57BL/6 mice in vivo. The results may be utilized in later stages as a method for the treatment of melanoma and the development of microRNAs-based therapeutic strategies for cancer treatment.

## Materials and methods

### Cell culture

The B16F10 melanoma skin cells were obtained from the National Cell Bank of Pasteur Institute (Tehran, Iran). They were cultured in DMEM (BioIdea, Iran), supplemented with 4.5 g/L of glucose, 10% FBS (BioIdea, Iran), 4 mM glutamine, 100 μg/mL of penicillin, and 100 μg/mL of streptomycin (Invitrogen, USA), and incubated in a humidified incubator at 37 °C. Afterwards, the cells were washed and detached with 0.25% trypsin/0.03% EDTA (BioIdea, Iran). Once 80% confluency was reached, the cells were seeded in culture plates at 30% confluency to be in the exponential growth phase.

### Cell transfection

The Mmu-miR-21a-5p sequence of nucleotides was obtained from www.MiRbase.org. Mouse miR-21 (accession No: MIMAT0000530) and nucleotide sequence of 5-UAGCUUAUCAGACUGAUGUUGA-3 are homologous between mice and man. Exiqon Co. (Copenhagen, Denmark) provided oligonucleotides for mmu-miR-21-5P, called miRCURY LNA inhibitor® (LNA-anti-miR-21), as well as its negative control, microRNA inhibitor negative control A (scrambled LNA). Labeling of two nucleotides was done at 5′ ends with fluorescein. The MiRCURY LNA inhibitor sequence is 5′-3′/56 FAM/ACA TCA GTC TGA TAA GCT and microRNA inhibitor negative control A (scrambled) sequence is 5′-3′/56-FAM/GTG TAA CAC GTC TAT ACG CCC A. LNA scrambled and LNA-anti-miR-21 were used at a final concentration of 50 μm. For transfection of LNA scrambled and LNA-anti-miR-21 to the B16F10 cell line, lipofectamine 2000 transfection kit (Invitrogen, USA) was used. Cells (2 × 10^5^) were cultured in 2 mL of high-glucose DMEM, containing 10% serum in each 6-well plate (SPL Life Sciences, Korea). The plate was placed in an incubator for 24 h at 37 °C. Next, the medium was washed completely in each well. After washing the cells with PBS once, the serum and antibiotic-free medium (1.8 ml) was added to the wells. Following that, LNA scrambled and LNA-anti-miR-21 (2 μL) were separately added to the transfection reagent (5 μL) in Opti-MEMI (200 μL; Gibco). Next, they were incubated for 5 min by pipetting and slowly mixed at room temperature for 20 min in darkness. After adding the collected complex to each six-well plate, cells were incubated for 10 h to allow transfection. Next, 200 μL FBS (10%) was added to the plate after 10 h of transfection to sustain maximum cell viability; next, it was incubated for 24 h. In addition, we cultured untreated cells (Control) and transfected cells with scrambled LNA (negative controls) along with LNA-anti-miR-21. The efficiency of transfection was examined by flow cytometry (Becton–Dickinson Immunocytometry System, USA) and fluorescence microscopy (Olympus, Tokyo, Japan). For in vivo studies, considering that the therapeutic effects of miR were desired and for comparing with the effects of LNA, the unmodified sequence of the anti-miR with a microsphere compatible with the in vivo environment was used.

### miRNA expression

For evaluating the feasibility of miR-21 inhibition by LNA-anti-miR, reverse transcriptase (RT)-PCR of microRNAs was carried out. After a day of transfection, FavorPrep™ Blood/Cultured Cell Total RNA Kit (Favorgen Biotech Co., Australia) was used based on column chromatography for the extraction of total RNA from the B16F10 cell line. A Nanodrop Epoch (BioTek, USA) was also used to measure the concentration of total RNA. Also, to synthesize cDNA from miRNA, cDNA synthesis kit (Exiqon, Denmark) was employed. To enhance cDNAs from miRNAs and detect miR-21 inhibition, BIOFACT™ 2X PCR master mix (BioFACT™, High ROX, Korea) was used in real-time PCR, along with miR-21-5P and miR-16 specific primers for normalization of real-time PCR; Exiqon (Denmark) provided all primers. Moreover, for 40-cycle real-time PCR, we used StepOnePlus real-time PCR system (Applied Biosystems, USA) was used. Finally, the relative miR21 expression was measured based on 2^−ΔΔCT^ method.

### Quantitative assays of apoptosis and necrosis

Apoptosis and necrosis were identified using the eBioscience V-fluorescein isothiocyanate apoptosis kit (V-FITC, USA). Briefly, 24 h after transfection, we examined the cells. After washing the plate-adhered cells with cold PBS, they were removed from the plate bottom using a scraper with gentle movements. The suspension was then added to flow cytometric tubes, and centrifugation was performed for 4 min at 190 g/4 °C. The cells were incubated for 10 min at 4 °C in an incubation buffer (100 μL), containing 2.5 μL of Annexin V and 5 μL of propidium iodide suspensions in the dark. The cells were then washed gently and analyzed by a FACSCalibur flow cytometer (Becton–Dickinson Immunocytometry System, USA) at an excitation wavelength of 488 nm with a 515 nm bandpass filter for Annexin-V (fluorescein-conjugated) detection and a 600 nm filter for PI detection.

### Assay for cell viability

After seeding 5 × 10^3^ cells in each 96-well plate, they were incubated in 5% CO_2_ at 37 °C. 24 h after transfection, the MTT assay was carried out. Following the addition of 12 mM MTT solution (10 μL; Bio-IDEA, Tehran, Iran) to each well, incubation was performed for 3 h at 37 °C. Next, the medium containing MTT was removed from the cells, and 100 μL of DMSO was added to each well and pipetted several times to form a completely uniform suspension. The solution was then shaken for 10 min at 37 °C until formazan crystals were completely dissolved. The obtained solution was added to a microtube and centrifuged in 1500 RPM for 5 min to eliminate any impurities. An ELISA reader was used to measure absorbance at 570 nm. For instance, all the mentioned steps were performed without adding the cells. The color concentration and percentage of cell survival were finally determined.

### In vivo experiments

Male C57BL/6 mice (20–30 g) aged 6–8 weeks were obtained from the Pasteur Institute. The animals had access to water and standardized pellet foods. We adhered to NIH guidelines in the design of all protocols. The mice were kept in the Animal Center for Physiological Research under pathogen-free conditions. The light conditions were also adjusted for 12 h of darkness and 12 h of brightness at 23 ± 2 °C. All mice were kept in the same environment at the animal house for 1 week prior to the experiments for acclimatization to the environment.

### Tumor development

After 1 week of acclimatization, B16F10 cells were detached with trypsin-EDTA from the flask after reaching 70% confluence and adjusted at 10^7^ cells/mL. Then, using an insulin syringe, each mouse received 100 μL of B16F10 cell suspension in the right flank in the subcutaneous space. In all mice, primary tumor cells appeared on the 7th day. On the 10th day, the mice were allocated to four groups (10 mice each). Following the inoculation of tumor, the mice were treated with intratumoral (IT) or intraperitoneal (IP) anti-miR on the 10, 13, and 16th days. On the 19th day, the animals were euthanized by IP injection of pentobarbital at a concentration of 60 mg/mL at a dose of 15 mg. Tumor was completely removed and weighed on a digital scale.

### Measurement of tumor volume

We determined the greatest longitudinal and transverse diameters of the tumor in order to measure its volume using an external caliper. Tumor volume was measured every 5 days by caliber measurement as follows:

$${\text{Tumor}}\,{\text{volume}} = {\raise0.5ex\hbox{$\scriptstyle 1$} \kern-0.1em/\kern-0.15em \lower0.25ex\hbox{$\scriptstyle 2$}}({\text{length}} \times {\text{width}}^{2} )$$

### LNA-anti-miR-21 in in vitro study

For transfection, a neutral lipid emulsion (MaxSuppressor™ in vivo RNA-LANCErII, BIOO Scientific) was used, as outlined by the manufacturer. 10 days after cell injection into the flank area, mice with confirmed tumor formation were divided into four groups of 10:

Group 1: melanoma mice to which PBS containing lipid amyloid was injected intratumorally;

Group 2: melanoma mice to which PBS containing lipid amyloid was injected intraperitoneally;

Group 3: melanoma mice to which LNA-anti-miR-21 lipid emulsion was injected intraperitoneally; and Group 4: melanoma mice to which lipid emulsion along with LNA-anti-miR-21 was injected intratumorally.

Considering the half-life of microspheres in blood, the mice were injected intraperitoneally and intratumorally with 200 μL of LNA-anti-miR-21 at a concentration of 50 nM once every 2 days. For examining the effect of LNA-anti-miR-21, the tumor was removed 9 days after the first injection when signs of physical weakness (shortness of breath, weight loss, and inability to move) appeared in the control group.

### Detection of miRNAs in tumors

Inhibition of miR-21 by LNA-anti-miR-21 was determined using RT microRNA real-time PCR. Nine days after the first LNA-anti-miR-21 injection, total RNA extraction was performed using GeneJET RNA purification kit (Thermo Scientific, Lithuania) according to the instructions. RNA concentration was adjusted to 5 nm. To synthesize cDNA from miRNAs, a universal cDNA synthesis kit was used (Exiqon, Denmark). Also, to enhance cDNA from miRNA and detect the amount of miR-21, a BIOFACT™ 2X real-time PCR master mix (BioFACT ™, High ROX, Korea), along with miR-21-5P and miR-16-5p specific primers, was used ( Exiqon, Copenhagen, Denmark) as internal control to normalize real-time PCR. We also used a StepOnePlus PCR system (Applied Biosystems, USA) for 40-cycle real-time PCR experiments, while 2^−ΔΔCT^ method was employed to calculate the relative miRNA-21.

### Quantitative PCR for mRNA detection

Total RNA was extracted from melanoma mice (injected melanoma cells into the Intratumoral and intraperitoneal tissues) and normal tissues using GeneJET RNA purification kit (Thermo Scientific, Lithuania) for the assessment of *SNAI1, Nes, Oct-4*, and *NF-kB* expression. In addition, a Nanodrop spectrophotometer (Thermo Scientific, USA) was used to measure total RNA. Briefly, Total RNA reached an exact concentration of 50 ng/μL. We used a cDNA synthesis kit (Thermo Fisher Scientific, Lithuania) to synthesize cDNA from single-stranded RNA; for real-time PCR, first strand cDNA was used as the template. Real-time PCR was used to measure the expression levels of *NF-kB, SNAI1, Oct-4*, and *Nestin* genes in tumor tissues. It was performed using BIOFACT 2X Real-Time PCR Master Mix (Korea). *SNAI1*, *Nes, Oct-4, NF-kB*, and *GAPDH* primers were prepared. Primer sequences of *Snai1* included 5′-GCGTGTGTGGAGTTCACCTT-3′ (forward) and 5′-CCAGGAGAGAGTCCCAGATG-3′ (reverse). Also, primer sequences of *POU5F1* (also known as *Oct-4*) were 5′-GGCGTTCTCTTTGGAAAGGTGTT-3′ (forward) and 5′-AAGGTTCTCATTGTTGTCGGCTTC-3′ (reverse), primer sequences of *NF-kB1* were 5′-ACACGAGGCTACAACTCTGC-3′ (forward) and 5′GGTACCCCCAGAGACCTCAT-3′ (reverse), and primer sequences of *Nestin* (*Nes*) were 5′-TGGAAGTGGCTACATACAGGACTC-3′ (forward) and 5′-GAGAAGGATGTTGGGCTGAGGA-3′ (reverse). *GAPDH* was used as the internal control (forward, 5′-GTAGCCATATTCATTGTCATA-3′; reverse, 5′-AATGCATCCTGCACCACCAA-3′). A qPCR cycler (Corbett Rotor-Gene 6000; Qiagen Corbett, Germany) was used for 40 cycles in real-time PCR, and comparative expressions of *Snail1*, *Nes*, *Oct-4, NF-ΚB1*, and *GAPDH* were calculated by 2^−ΔΔCT^ method. The cycling conditions included 10 min of initial denaturation at 95 °C and 40 cycles of denaturation at 95 °C for 15 s and at 60 °C for 60 s (annealing and extension). SPSS V. 23.0 (USA) was used for data analysis.

### Immunohistochemistry of *CD133* and *NF-kB* in tumor samples

The tumors were removed and fixed in 10% formalin. Following tissue preparation, paraffin blocks were sectioned using a Leitz 15/2 microtome. Based on the standard IHC protocol, mouse monoclonal antibody was used to stain tumor tissue samples against *CD133* (Cat # ab16518; Abcam) and *NF-kB* (Cat # sc-8008; Santa-Cruz). Next, xylene was used to deparaffinize paraffin-embedded Sects. (5 µm). Also, 3% H_2_O_2_ was used to block endogenous peroxidase activity in methanol for half an hour. After dehydrating the tissue sections with graded alcohol, antigen retrieval was achieved using 10 mM sodium citrate. Next, TBST was used to wash the sections, while 5% bovine serum albumin (BSA) was used for blocking in 1 h.

Slides were incubated with the mouse monoclonal antibody against *NF-kB* and *CD133* (diluted in TBS at 1∶50). After washing the slides in TBST for 5 min, they were incubated with HRP-conjugated anti-mouse antibody, which was diluted in TBS (1∶200). The slides were incubated with DAB (Sigma) after TBS washing; following color development, they were rinsed with tap water immediately. All the slides were stained with haematoxylin, after which they were observed under a light microscope. Imaging was performed using an optical microscope (BX51, Japan), connected to a camera (DP12, Japan). The positive cell count in 10 fields of 10 sections was obtained using Image J (version 1.48), based on the scale bar set on images, with optical microscope adjusted on the measurement scale.

### Statistical analysis

In this study, experiments were performed in triplicate, and data are presented as mean ± SD. For comparing the groups, Kruskal–Wallis test was carried out. SPSS version 23 was used for data analysis. *P < 0.05 and **P < 0.01 were considered significant.

## Results

### Expression of miR-21 in B16F10 cells

**Fig. 1:**
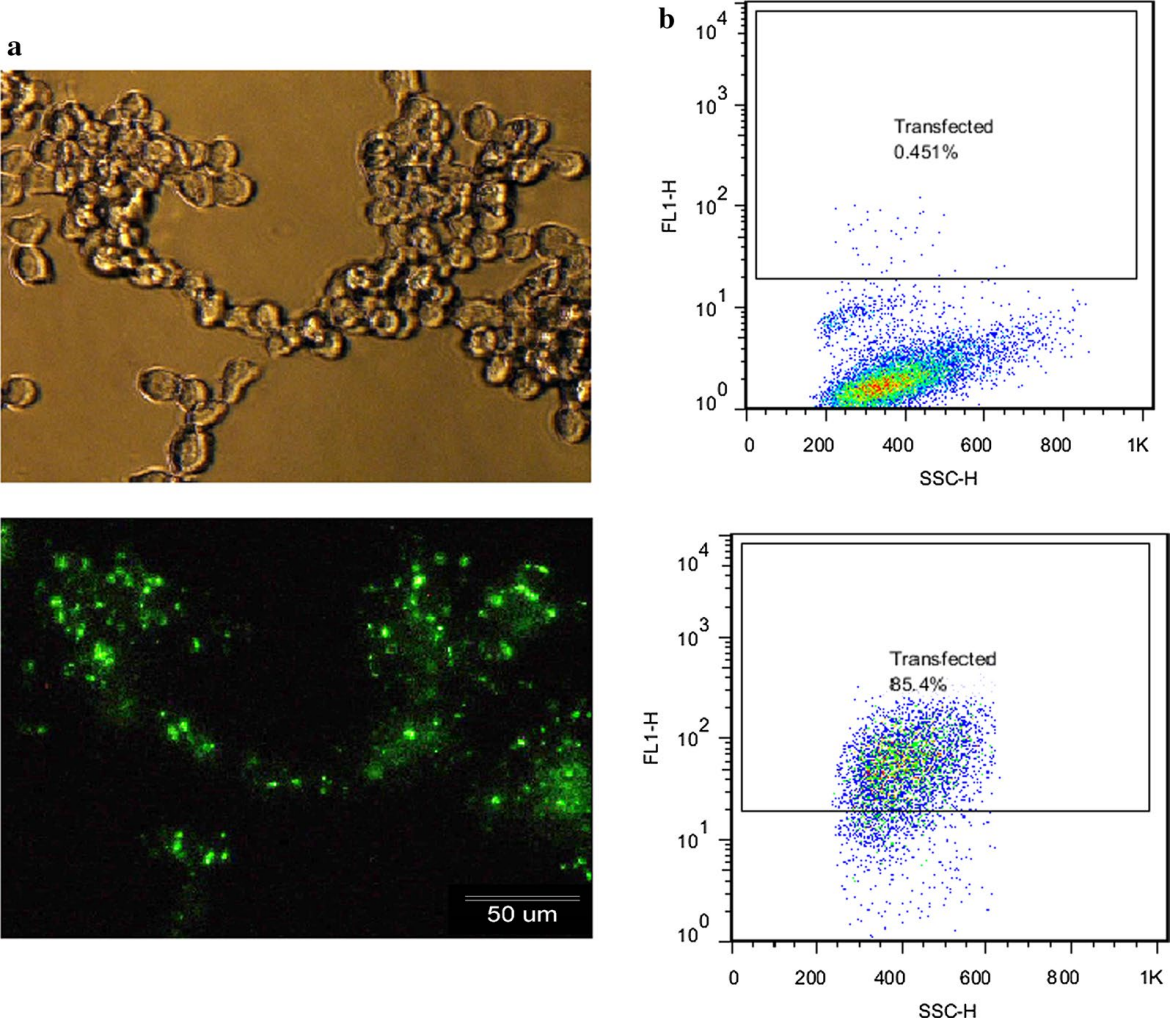
Evaluation of transfection efficiency in B16F10 cells using a FAM-labeled scrambled LNA: Control compared with scrambled LNA transfected cells, **a** Fluorescence microscopy detected green fluorescent cells. **b** Flow cytometry showed more than 80% transfected cells

**Fig. 2:**
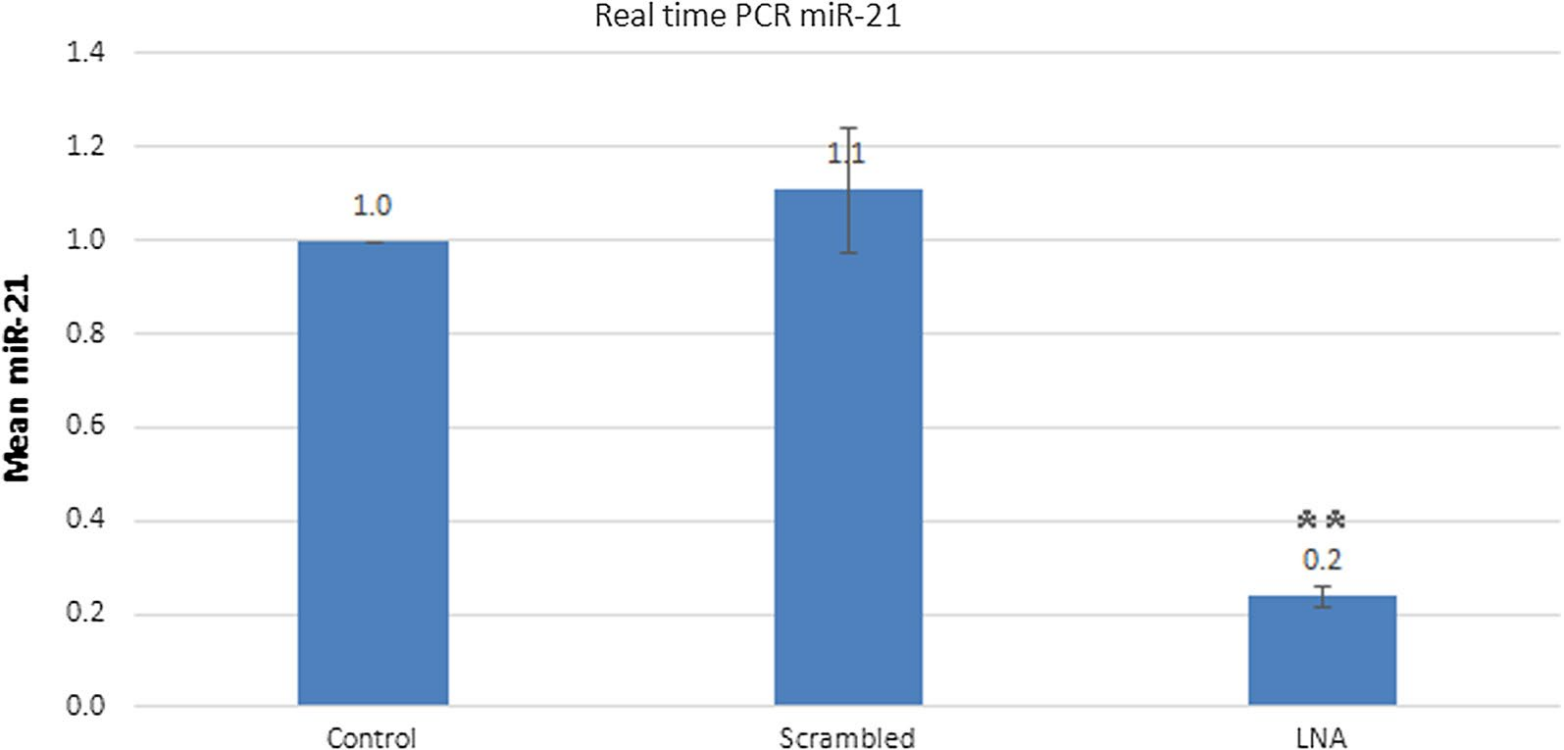
Evaluation of the expression of miR-21, 24 h after transfection using 2^−ΔΔCt^ method by real-time PCR. The control group was used as the reference for comparison with other groups

For the miR-21 inhibition assay in B16F10 cells, transfection efficiency was initially performed in vitro using FAM-labeled scrambled control and lipofectamine 2000 to enter RNA oligos into human B16F10 cells. Afterwards, fluorescence microscopy and flow cytometry were used to determine the transfection efficiency. Green fluorescent signals were found in more than 80% of cells (Fig. 1). After ensuring the efficiency of transfection, changes in miR-21 expression in untreated cells and cells transfected with LNA scrambled and LNA-anti-miR-21, using 2^−ΔΔCT^ method by real-time RT-PCR miRNA. This analysis showed that the expression of miR-21 was related to the LNA-anti-miR-21 group, and miR-21 did not significantly differ from untreated cells regarding scrambled LNA transfected cells (*P* > 0.01). In all three repetitions, the expression of miR-21 was significantly lower in the LNA-anti-miR group compared with scrambled LNA and untreated groups (**P < 0.01) (Fig. 2).

### MiR-21 inhibitor decreased viability in B16F10 cells

**Fig. 3:**
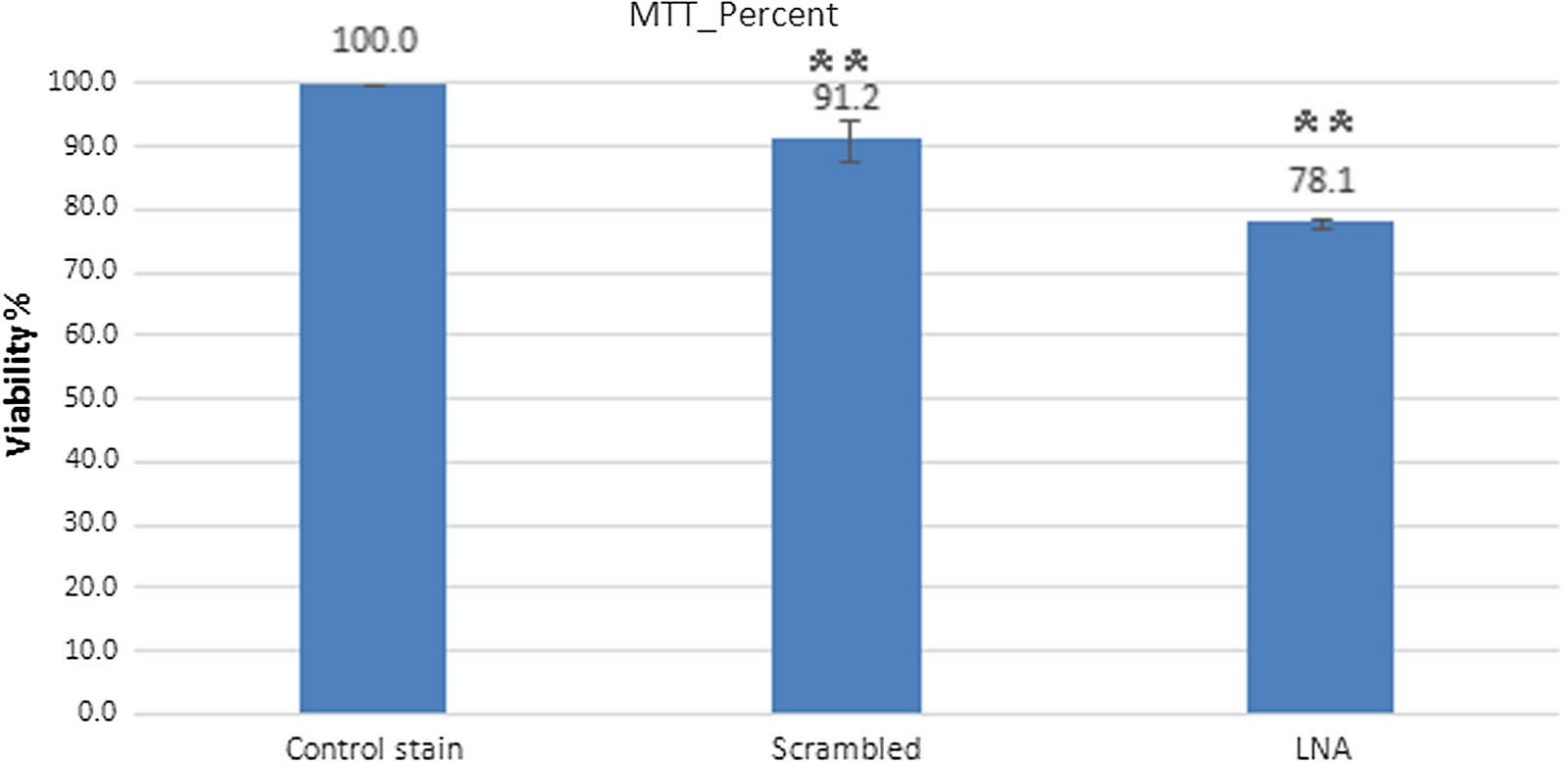
LNA-anti-miR-21 caused a significant reduction in B16F10 cell viability compared to the scrambled and control groups (**P < 0.01)

The MTT assay was applied to determine cell viability in miRNA inhibitor-transfected B16F10 cells 24 h after transfection. In the LNA-anti-miR-21 group, viability of B16F10 cells was lower than scrambled LNA and control groups after 24 h (*P* < 0.01). The findings showed that LNA-anti-miR-21 decreased B16F10 cell viability (scrambled LNA: 91.1967 ± 3.16; LNA-Anti-miR-21: 78.1333 ± 0.76; and control: 100 ± 0.0%). These findings indicate that inhibition of the function of miR-21 reduced the viability of melanoma cells (Fig. 3).

### MiR-21 inhibitor induces apoptosis in B16F10

**Fig. 4:**
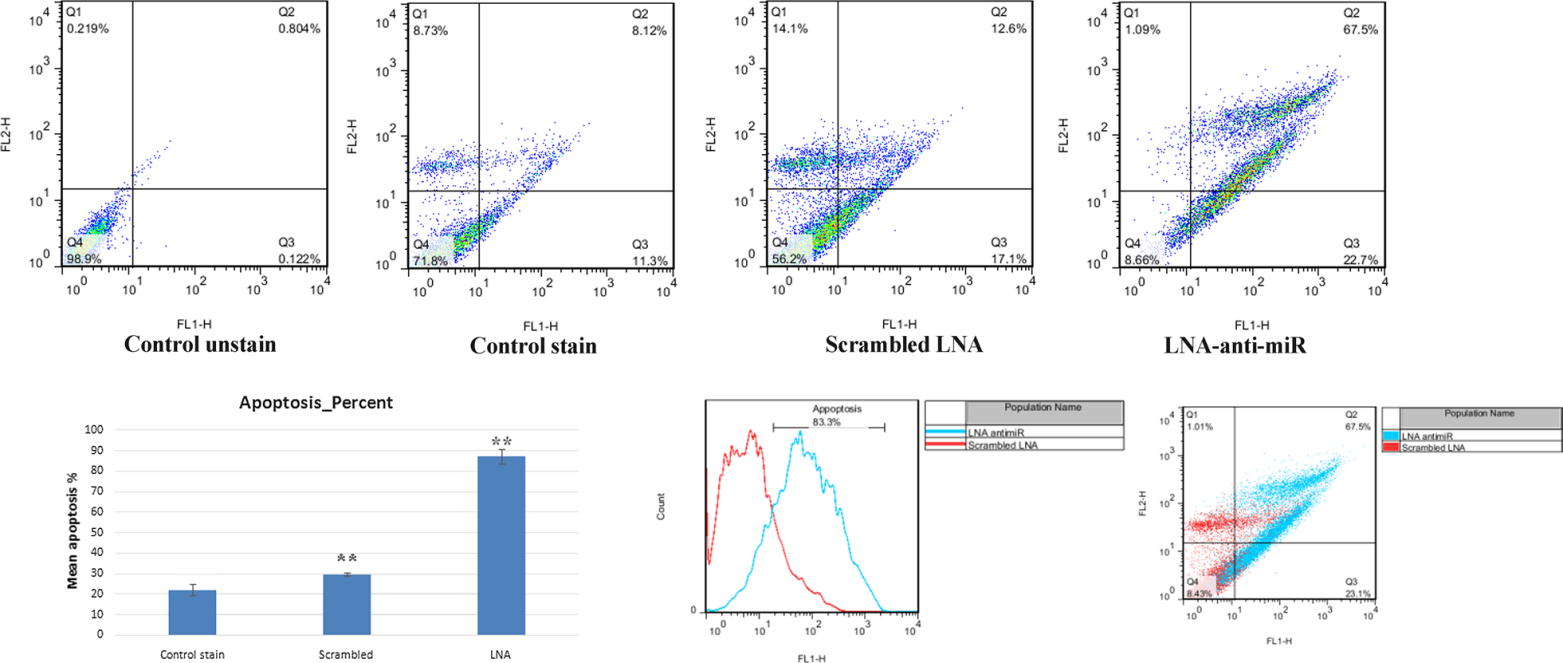
LNA-anti-miR-21 has a significant effect on apoptosis percentage in comparison with untreated and scrambled LNA cells (**P < 0.01). The Figure indicates the apoptosis profile following annexin V/PI staining; LNA-anti-miR-21 increased apoptosis. The histogram indicates the percentage of apoptotic cells

Annexin V is a phosphatidyl serine-binding protein in the presence of calcium ion; hence, it is very suitable for the detection of apoptotic cells in a cell population. PI allows for the detection and purification of necrosis cells. The apoptotic cell percentage, positive with Annexin V, was higher in transfected cells with LNA-anti-miR-21, compared with untreated cells and those transfected with scrambled LNA (scrambled LNA: 29.800 ± 0.89; LNA-anti-miR-21: 87.2867 ± 3.57; and control: 22.0833 ± 2.69%). LNA-anti-miR-21 transfection, which inhibited miR-21, significantly increased B16F10 cell apoptosis (Fig. 4).

### LNA-anti-miR-21 reduced miR-21 in the in vivo study

**Fig. 5:**
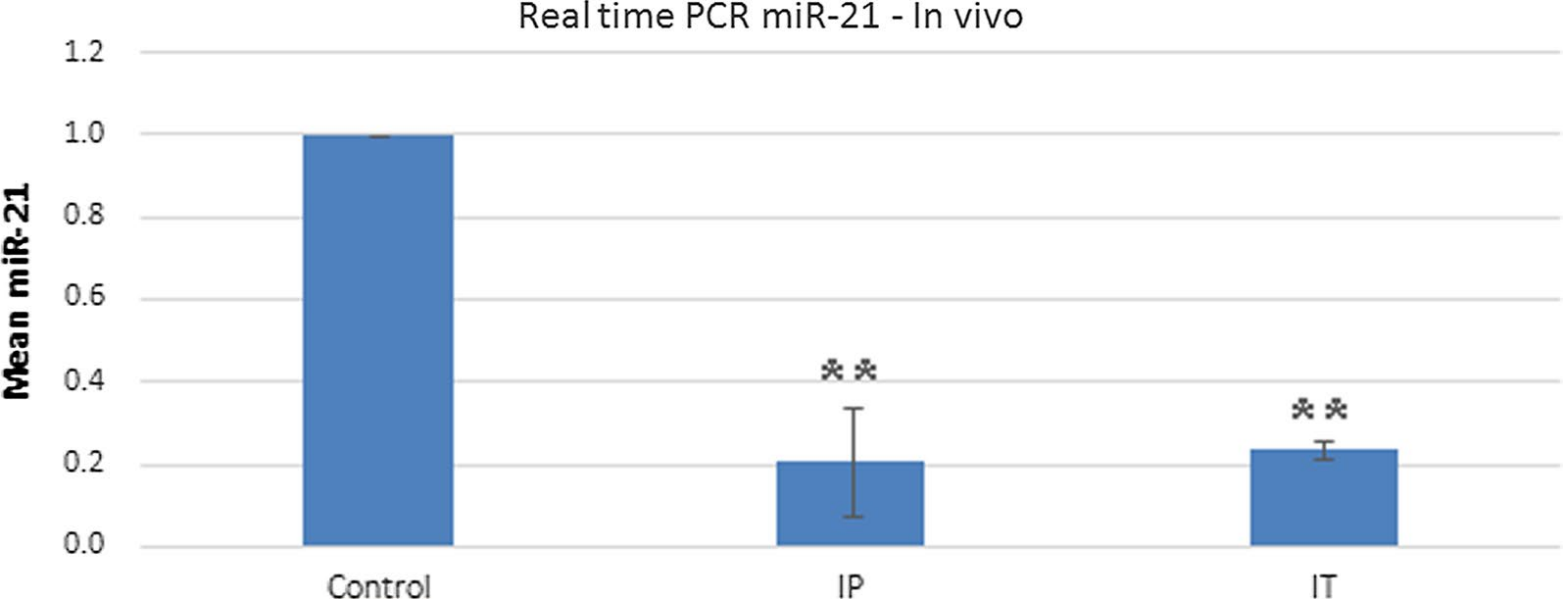
Expression levels of miR-21 in both IP and IT performed 24 h following transfection, using 2^−ΔΔCt^ method by real-time PCR. The untreated group was used as the control for comparison with other groups

Based on real-time PCR assay of mouse tumors, miR-21 expression was significantly lower in tumors treated with anti-miR than untreated tumors in both IP and IT injections. The inhibition level of miR-21 was the same in both groups (IP: 0.2083 ± 0.6768; IT: 0.2357 ± 0.05662; and control: 1 ± 0.0) (Fig. 5).

### LNA-anti-miR-21 modulated *SNAI1, Nes, Oct-4 and NF-kB* expression in mouse tumor genes of anti-miR inhibited cells

**Fig. 6:**
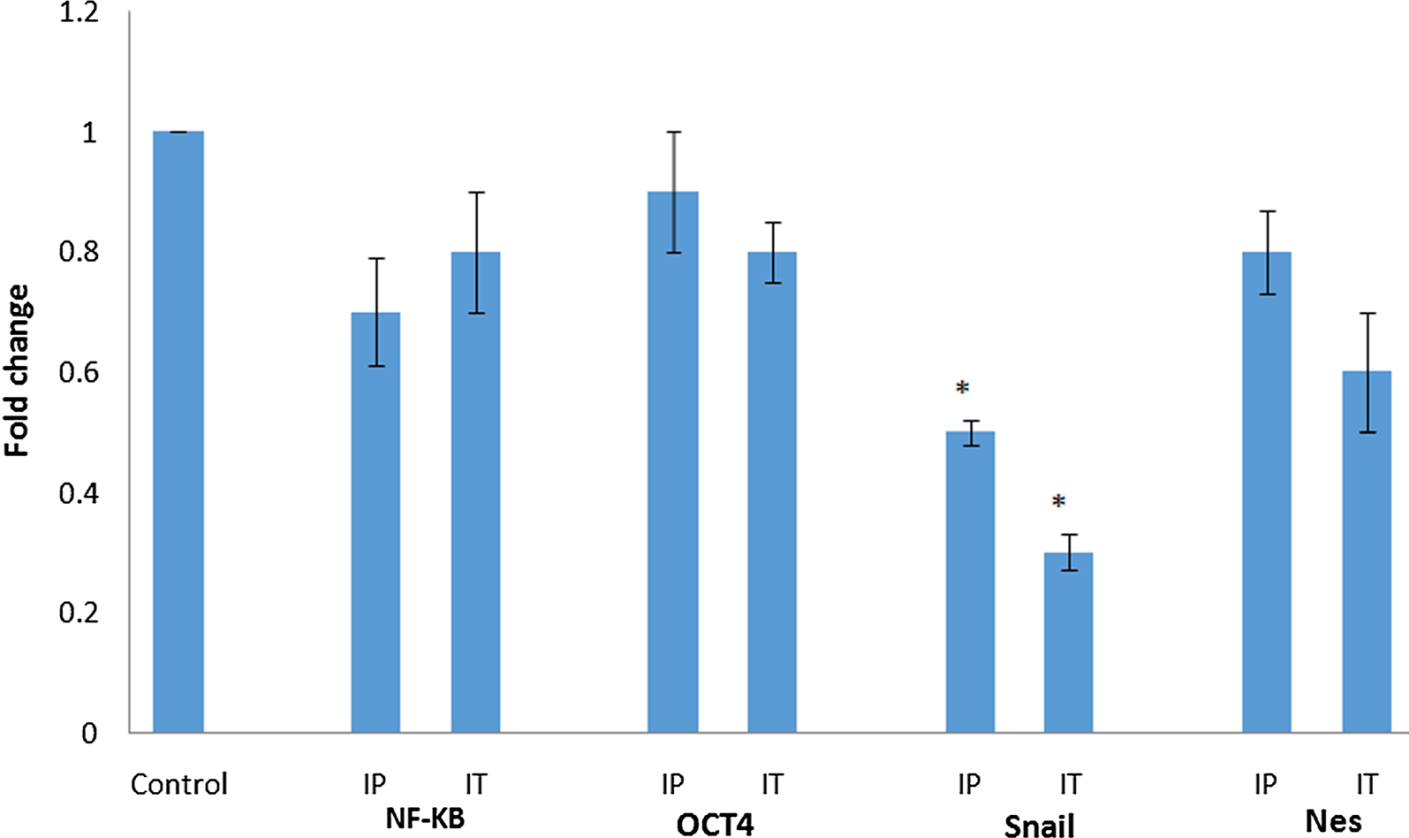
Expression levels of *Snai1, Nes, NF-kB*, and *NF-kB* mRNA after 19 days of LNA-anti-miR-21 injection by 2^−ΔΔCt^ method using real-time PCR. The expression level of *SNAI1 *in cells treated with LNA-anti-miR-21 decreased significantly

Using real-time PCR, the expression patterns of *SNAI1, Nes, Oct-4*, and *NF-kB* were specified. The expression level of *SNAI1* in cells treated with LNA-anti-miR-21 decreased significantly. The mRNA expression level of other genes reduced, though not significantly compared with the untreated groups (Fig. 6).

### Tumor volume measurement

**Fig. 7:**
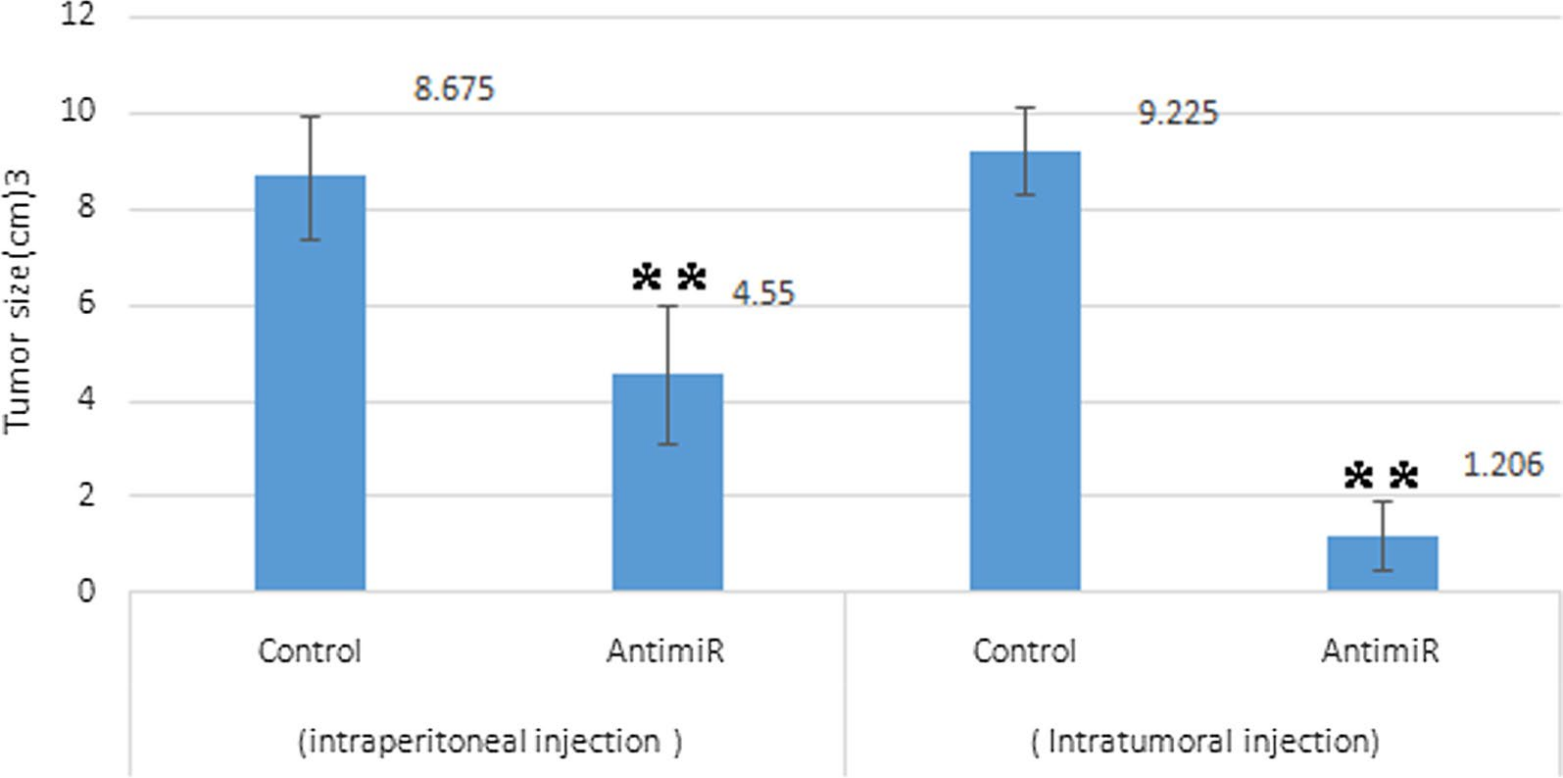
Effect of LNA-anti-miR-21 on tumor volume compared with the control group. The highest longitudinal and transverse diameters were determined to measure tumor volume (**P < 0.01 versus the controls). It seems that IP and IT LNA-anti-miR-21 injection has a greater effect on inhibiting tumor size

Tumor volume reduced in tumors injected with IP and IT LNA-anti-miR-21 in comparison with the control group (significant in IP and IT injections). It seems that IP LNA-anti-miR-21 injection has a greater effect on inhibiting tumor size (Fig. 7). The delivery of IP and IT of LNA-anti-miR created the same degree of in vivo downregulation of miR-21, the suppression of xenograft growth correlated with the IP and IT route. These findings indicated that LNA’s anti-melanoma impact was due to miR-21 inhibition in the tumor cells.

### Immunohistochemistry of cd133 and NF-κB protein

**Fig. 8:**
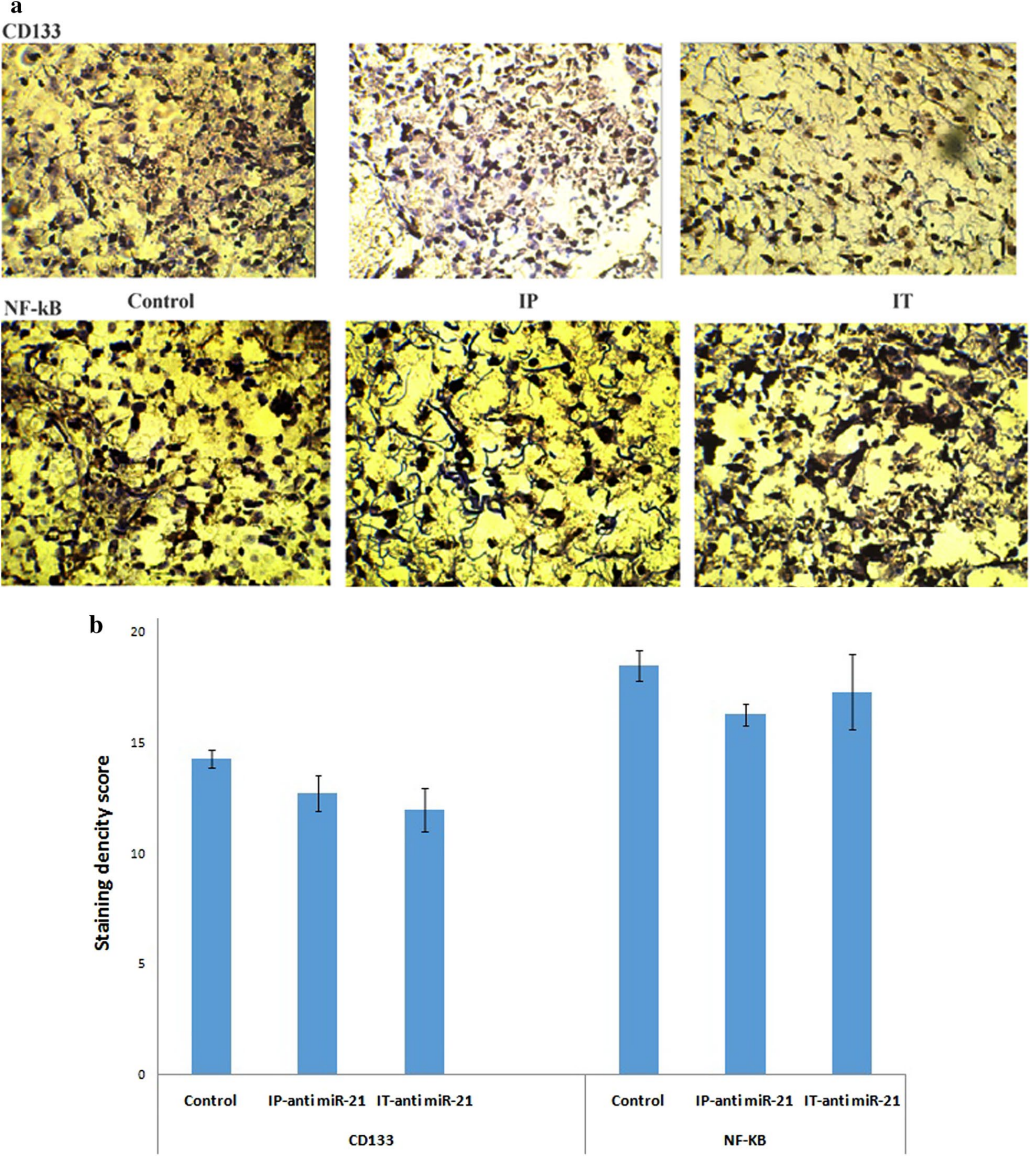
**a** Immunohistochemical evaluation of *CD133* and *NF-kB*. Images are tumor staining fields for each treatment. The low-power scale bar is 2 μm, and the high-power scale bar is 50 μm. **b** Quantification of melanoma tumors data compared to control (untreated cells). The levels of CD133 and *NF-kB* proteins reduced, though not significantly compared to the control cells *P < 0.05 and **P < 0.01

In investigations on tumor tissue samples treated with IP and IT injections of LNA-anti-miR-21, the levels of CD133 and NF-kB proteins reduced, though not significantly compared to the control cells (*P < 0.05 and **P < 0.01) (Fig. 8).

## Discussion

Abnormalities in the activity and function of miRNAs lead to the development and progression of various types of cancers. Therefore, it is not surprising that miRNAs have attracted the attention of tumor specialists and have been considered as new diagnostic, medical, and therapeutic markers [19]. Other studies have shown that miRNAs play a fundamental role in many disorders, including cancerous, rheumatologic, inflammatory, cardiovascular, autoimmune, and metabolic diseases [20–22]. Overexpression of certain miRNAs can act as an oncogene due to the negative regulation of tumor suppressor genes, differentiation of controller genes, or cell apoptosis. Other expressions, however, may act as a tumor suppressor through negative regulation of oncogenes or genes involved in non-differentiation or anti-apotheosis. Today, it is recognized that miRNAs can also act as an oncomiR in cancers [11]. For melanoma cancer, chemotherapy, radiotherapy, and surgery are used as the primary treatments. However, most patients require a combination therapy. Surgical removal or surgical treatment of primary tumors has to date been the principal treatment option. Chemotherapy was then introduced. However, metastatic melanoma is usually resistant to commonly used anticancer drugs [23]. Despite the fact that chemotherapy destroys most cancer cells, it can rescue tumor stem cells, which provide an important mechanism of resistance in excessive drug-resistant tumors. Therefore, anti-cancer strategies must be targeted at cell populations [7].

There are several pathways leading to drug resistance, such as apoptosis, excessive pro-survival signaling pathways, activation of alternative compensatory pathways, overexpression of therapeutic targets, increased level of heterozygosity, and overexpressed efflux pumps for drug withdrawal [24, 25]. Today, natural autoantibodies (Nabs) are considered for cancer treatment as a novel approach [26]. Therefore, it is necessary to develop new and more effective therapies for cancer treatment. There was reported to be a significant correlation between the increased expression levels of miR-21-5p and depth of invasion, cancer mitotic index, invasion of lymphovascular and AJCC stage [27]. In contrast, let-7b levels were significantly decreased in invasive and in situ melanomas compared with common and dysplastic nevi [27]. On the contrary, there was a significant correlation between decreased levels of let-7b and in situ melanomas in comparison with common and dysplastic nevi [27]. MiR-21, 204 and 125b may affect melanoma tumorigenesis [27]. The obtained results indicate that an increase in the expression of miR-21 leads to increased possibility of invasive melanoma cell lines via tissue inhibitor of the metalloproteinase inhibitor 3. Therefore, miR-21 inhibition in melanoma may decrease the invasion of melanoma.

LNA combinations have been recently investigated in various research phases. Researchers have reported that patients with early melanoma lesions and very high expressions of miR-199a-5p, miR-1908, or miR-199a-3p have a much shorter survival compared to patients with early melanoma and lower miRNA [28, 29]. The combined expression of these miRNAs has also shown a strong predictive potential with regard to the disease. Interestingly, the transfer of LNA-based cocktail drug caused a very severe metastasis reduction Therefore, combination of these three miRNA targets could be therapeutic for melanoma cancer [19]. Generally, escape from apoptosis is one of the most important changes in cellular physiology, which can increase tumor growth. Induction of apoptosis is the Achilles’ heel of new treatments; hence, apoptosis is one of the most important features of cancer control. The small size of miRNAs and their ability to regulate the growth of cancer cells conduce to apoptotic brake. The presence of a distinctive miRNA pattern in the body tissues and changes in this pattern due to diseases such as cancer suggest the impairment of miRNA expression in such diseases [18]. In this study, LNA-anti-miR-21 was used for miR-21 inhibition. The effects of this inhibition in vitro were investigated on proliferation, apoptosis and necrosis and cell viability of B16F10 cell line. In animal studies, the inhibitory effects on tumor volume, immunohistochemistry of NF-kB and CD133 markers, as well as expression of *Nes, Oct-4*, and *NF-kB* genes, have been investigated. In this in vitro study, we were able to transfect over 80% of the cells. Transfection efficiency was measured by fluorescence microscopy, and data were verified by flow cytometry. The results of real-time PCR showed the LNA-anti-miR-21 inhibition of miR-21 in a murine melanoma cell line (B16F10). The MTT assay of miR-21 inhibition on cell viability revealed that LNA-anti-miR-21 could decrease the proliferation of B16F10 cell line by 13%, compared to the scrambled LNA.

In comparison with the control and scrambled LNA groups, apoptosis had a significant increase in cells transfected with LNA-anti-miR-21 Also, LNA-anti-miR-21 transfected cells included fewer necrotic cells compared to the control and scrambled LNA groups. It seems that miR-21 inhibition increases apoptosis in comparison with necrosis, which is due to the ability of miR-21 as an oncomiR to prevent apoptosis through inhibiting mRNA tumor suppressor proteins. Low levels of necrosis compared with apoptosis indicate the capability of this method to destroy tumors in biological systems. Melanoma has a great potential for metastasis in a way that the primary tumor begins the metastatic outgrowth shortly after its beginning. Multiple factors can lead to high metastasis. The most important property of this type of tumor, superior to other tumors, is its stemness, the removal of which disrupts the growth and development of malignant tumors. Therefore, miR-21 can act as a protector of tumor cells whose inhibition induces apoptosis.

Considering the significant increase in apoptosis compared to necrosis in this study, LNA-anti-miR-21 may be used as a suitable candidate in combination with other therapeutic agents in the treatment of melanoma cancer. Even if the tumor is resistant to common treatments, it can be used to sensitize the tumor to other therapeutic agents. Moreover, by adopting a natural apoptosis process, compatible with the natural biology of cells, it can be used as a targeted therapy.

The expression of *SNAI1, Nes, Oct-4*, and *NF-kB* genes in cells inhibited by LNA-anti-miR-21 was further investigated in vivo. Among these genes, only *Snail* gene showed a significant decrease in comparison with the control group. The increased expression of *Snail* gene in many cancers, especially melanoma [30], and its effects on the migration of tumor cells, has made it an important marker in skin melanoma cancer. Recently, EMT has been shown to be associated with cancer stem cell phenotype [31–33]. *SNAI*is overexpressed in tumors obtained from human surgery. This increase in its expression creates EMT and similar cancer stem cell phenotypes in other cell lines [34]. In many studies, *Snail *gene inhibition was used to prevent the growth, invasion, and metastasis of tumors [35–37]. The delivery of IP and IT of LNA-anti-miR created the same degree of in vivo downregulation of miR-21. These findings suggested that IP and IT LNA delivery may be due to the systemic inhibition of miR-21. In the IP injection may be occurred in the immune cells that successively prevented the growth of xenograft.

Studies on tumor tissues have shown no significant reduction in the expression of CD133 and NF-ΚB markers. Regarding the size of tumors, when LNA-anti-miR was systemically injected, tumor volume reduced significantly in comparison with the control group.

## Conclusion

In vitro and in vivo studies show that miR-21 inhibition can effectively reduce the proliferation and increase the apoptosis of melanoma cells. Given the low levels of necrosis, LNA-anti-miR-21 uses the normal cellular pathway to destroy melanoma cells. Reducing the expression of *Snail* gene can conduce to the prevention of the development of cancer cell metastasis. Accordingly, anti-miR-21 may be potentially used as a novel therapeutic agent to eliminate melanoma cells. Finally, LNA-anti-miR-21 can be considered a novel therapeutic option for patients with skin melanoma cancer and high miR-21 expression.
